# TNFα-reliant FSP1 up-regulation promotes intervertebral disc degeneration via caspase 3-dependent apoptosis

**DOI:** 10.1016/j.gendis.2024.101251

**Published:** 2024-02-28

**Authors:** Cheng Qiu, Lin Cheng, Derun Di, Ziqian Xiang, Congyu Wang, Jinghang Li, Yinuo Xiong, Manyu Li, Jingwei Liu, Jian Zhou, Tianyi Liu, Xinyu Wang, Dan Luo, Xiaoxiong Wang, Shangye Li, Hui Wang, Xia Wang, Yunpeng Zhao, Xinyu Liu, Lianlei Wang

**Affiliations:** a Department of Orthopaedic Surgery, Qilu Hospital of Shandong University, Jinan, Shandong 250012, China; b Cheeloo College of Medicine, Shandong University, Jinan, Shandong 250012, China; c Department of Emergency Medicine, Qilu Hospital of Shandong University, Jinan, Shandong 250012, China; d Department of Gastroenterology, Qilu Hospital of Shandong University, Jinan, Shandong 250012, China; e Department of Pediatric Surgery, Qilu Hospital of Shandong University, Jinan, Shandong 250012, China; f Department of Orthopedics, The Second Xiangya Hospital of Central South University, Changsha, Hunan 410011, China; g Department of Medical Oncology, National Cancer Center/National Clinical Research Center for Cancer/Cancer Hospital, Chinese Academy of Medical Sciences and Peking Union Medical College, Beijing 100021, China; h Department of Molecular Orthopaedics, National Center for Orthopaedics, Beijing Research Institute of Traumatology and Orthopaedics, Beijing Jishuitan Hospital, Capital Medical University, Beijing 100035, China; i Department of Oral and Maxillofacial Surgery, The Affiliated Hospital of Qingdao University, Qingdao, Shandong 266003, China

**Keywords:** Caspase 3, FSP1, iFSP1, Intervertebraldisc degeneration, NF-κB, TNFα

## Abstract

Intervertebral disc degeneration (IDD) is a common chronic inflammatory degenerative disease that causes lower back pain. However, the underlying mechanisms of IDD remain unclear. Ferroptosis suppressor protein 1 (FSP1) is a newly identified suppressor for ferroptosis. This study aims to investigate the role of FSP1 in IDD. Nucleus pulposus (NP) tissues in humans were collected and NP cells from rats were isolated to detect FSP1 expression pattern. The relationship between FSP1-mediated ferroptosis and apoptosis was identified using FSP1 inhibitor iFSP1. RNA sequencing was utilized to seek downstream molecules and related signaling pathways. Moreover, both exogenous recombinant FSP1 protein and endogenous small interfering RNA were implemented in this study to clarify the role of FSP1 in tumor necrosis factor-alpha (TNFα)-mediated NP cell apoptosis. Ultimately, the underlying mechanisms of FSP1-related signaling pathway in IDD were uncovered both *in vitro* and *in vivo*. As a result, FSP1 was up-regulated in human degenerative NP tissues and after TNFα stimulation. FSP1 inhibition by iFSP1 fails to trigger ferroptosis in NP cells while inhibiting TNFα-mediated apoptosis. Further experiments demonstrated that FSP1 was closely related to TNFα-reliant caspase 3 activation and mitochondrial damage. However, the exogenous addition of recombinant protein FSP1 does not induce cell death or intensify the efficacy of TNFα. Mechanically, FSP1 is involved in TNFα-mediated NF-κB signaling activation to accelerate the development of IDD. This study demonstrated that FSP1 promotes IDD through TNFα-reliant NF-κB signaling activation and caspase 3-dependent apoptosis. These findings suggested a novel therapeutic target for the treatment of IDD.

## Introduction

Lower back pain is a common symptom in clinic that enormously affects the life quality of individuals and causes socioeconomic expenditures worldwide.^1^^,^^2^ According to statistics, more than 70% population around the world are suffering from chronic lower back pain, and 11% of patients with lower back pain are deprived of labor capability.^3^, ^4^, ^5^ Intervertebral disc degeneration (IDD) is the major cause of lower back pain thus resulting in a set of maladies containing disc herniation, spinal stenosis, and vertebra instability.^6^, ^7^, ^8^ IDD is an age-related degenerative musculoskeletal disease with uncertain pathogenesis, so only palliative therapeutic strategies such as pharmacological applications on pain relief are utilized to treat this condition.^9^^,^^10^ It is of great significance to expound on the molecular and pathogenic mechanisms to propose targeted approaches to the treatment of IDD.^11^

Nucleus pulposus (NP) cells with normal metabolism maintain the homeostasis of the intervertebral disc. Otherwise, their senescence and demise trigger IDD.^12^^,^^13^ Notwithstanding, it is recognized that the NP cells' senescence and demise are closely in line with persistent chronic inflammatory reactions.^14^ Chronic inflammation induced by proinflammatory cytokines like tumor necrosis factor-alpha (TNFα) during IDD is one of the major causes of metabolic disorder and dysfunction.^15^^,^^16^ The grade of IDD is in concordance with the expression level of TNFα.^17^^,^^18^ However, it is still revealed that multiple factors engage in cell aging and death, promoting NP cells succumbing to several types of cell death such as necroptosis, apoptosis, ferroptosis, pyroptosis, and autophagy.^19^, ^20^, ^21^, ^22^, ^23^, ^24^ Apoptosis in IDD is well-established and its interruption gains optimal efficacy of preventing the degeneration process.^25^ Instead, ferroptosis is a novel form of cell death with the manifestation of iron-overloading lipid peroxidation, and it has been corroborated in various pathophysiological changes.^26^, ^27^, ^28^, ^29^, ^30^ Several studies imply that ferroptosis is involved in cell death of intervertebral disc thus mediating the progression of IDD. It is concluded that glutathione peroxidase 4 (GPX4), ferritin heavy chain, and ferroportin are the specific defenses to separately combat tert-butyl hydroperoxide-induced acyl-CoA synthetase long-chain family member 4 (ACSL4)-reliant lipid peroxidation as well as ferric ammonium citrate induced excessive iron.^22^^,^^31^, ^32^, ^33^ Whereas the exploration of ferroptosis in IDD is in its infancy and many unknowns remain to be uncovered, corroboration of molecular therapeutic targets on IDD might benefit from relevant breakthroughs.

Ferroptosis suppressor protein 1 (FSP1), also named as apoptosis-inducing factor mitochondria-associated 2 (AIFM2) and apoptosis-inducing factor (AIF)-homologous mitochondrion-associated inducer of death (AMID), is renominated for lipid radical scavenging by regeneration of reduced coenzyme Q_10_ against ferroptosis in the absence of GPX4.^34^, ^35^, ^36^ So akin to AIFM1, FSP1 is firstly recognized as a pro-apoptotic inducer that translocates into nuclei to mediate caspase-independent cell death.^37^^,^^38^ However, FSP1 is different from AIFM1 due to the lack of a mitochondrial localization sequence whose function is to determine the protein location referred to mitochondria, and therefore a majority of FSP1 adheres to the outer membrane of mitochondria, whereas the other part of FSP1 resides in the cytoplasm and rare in nucleus.^34^^,^^39^ Thereunder, elevation of FSP1 and the subsequent location change could trigger cell apoptosis.^40^^,^^41^ Nevertheless, up-regulation of FSP1 to combat ferroptosis is advantageous to cell survival as revealed by recent studies.^42^^,^^43^

Herein, it is still unknown how FSP1 functions between apoptosis and ferroptosis during IDD, and the underlying mechanisms need to be clarified. The objective of this study is to elucidate the potential role of FSP1 in IDD.

## Materials and methods

### Human ethics statement

The study was approved by the Institutional Review Board Committee of Qilu Hospital of Shandong University (number: KYll-2021 (ZM)-058) and was conducted in accordance with the Declaration of Helsinki. From July 2021 to November 2022, 25 patients (15 males and 10 females; 3–76 years old; mean age = 40.76 ± 21.51 years old) who experienced orthopedic surgery at Qilu Hospital of Shandong University were enrolled and signed informed consent. They suffered from disk herniation, spinal stenosis, spondylolisthesis, spinal trauma, hemivertebrae, isthmic spondylolisthesis, and spondyloptosis separately, as diagnosed by experienced doctors. The intervertebral discs were obtained intraoperatively. According to the Pfirrmann grading system, the distinct degenerative grade of intervertebral disc (detailed information was presented in Table S1) was distinguished by magnetic resonance imaging (MRI) and classified into two groups (Grade I–II and Grade IV–V).

### Animal ethics declaration

All animal procedures were approved by the Laboratory Animal Centre of Shandong University. Six-week-old, eight-week-old, and sixteen-week-old Sprague–Dawley rats were purchased from the Animal Center of Shandong University and were used in this study. All rats were housed under identical specific pathogen-free standard environmental conditions (23 °C ± 2 °C, 12 h light/dark cycle) with free access to food and were allowed to move freely.

### Rat IDD model

Sixteen-week-old rats were used to establish the coccygeal vertebral needle puncture model.^44^ All rats were randomly divided into three groups, Blank group, PBS group, and iFSP1 group. Briefly, after anesthetization with isoflurane, the area between the eighth and ninth coccygeal vertebrae (Co8–Co9) of Sprague–Dawley rats was palpated and then punctured by a 20-gauge needle. The Blank group was untreated, the PBS group was needle punctured and injected with PBS, and the iFSP1 group was needle punctured and injected with 10 μM iFSP1.

### Rat NP cells

Six-week-old rats were used for the isolation of primary coccygeal vertebral NP cells. Firstly, rats were sacrificed by cervical vertebra dislocation and immediately soaked in 75% ethyl alcohol for 10 min. Then the whole tail was detached and coccygeal NP tissue was harvested under sterile conditions. Total discs were isolated and submerged in a culture medium with Hanks' balanced salt solution. The NP tissue was placed into a 15 mL tube containing 0.2% type II collagen (Sigma–Aldrich, St. Louis, USA) under 37 °C for 4 h. Rat NP cells were acquired after centrifugation and cultured in DMEM/F-12 medium (HyClone, Thermo Co., USA) supplemented with 10% fetal bovine serum (Gibco, USA), 1% 100 U/mL penicillin, and 100 mg/mL streptomycin (HyClone, USA). All cells were cultured in an incubator at 37 °C under 5% CO_2_. The culture media were replaced after rat NP cell adherence. Later, the culture media were replaced every 2 days, and the cells were passaged when they reached 80%–90% confluence.

### RNA sequencing

Total RNA was extracted using RNAprep Pure Plant Plus Kit (DP441, TIANGEN) and purified by RNAClean XP Kit (A63987, Beckman Coulter) and RNase-Free DNase Set (79,254, Qiagen). Then the mRNA library construction and high-throughput sequencing were performed by Hangzhou KaiTai Biotechnology Co., Ltd. Libraries were constructed using U-mRNAseq Library Prep Kit (AT4221, KAITAI-BIO) with Ribo-off rRNA Depletion Kit (Bacteria) (N407, Vazyme). Libraries were pooled and sequenced using the Illumina NovaSeq machine as 150-bp paired-end sequencing reads.

### Western blot

To extract the protein, a high-efficient RIPA lysis buffer (R0010, Solarbio, China) containing 1 mM PMSF was added. Protein electrophoresis was carried out on a 12% SDS-PAGE gel, and the proteins were electroblotted onto nitrocellulose membranes. Then the membrane was blocked in 5% non-fat dry milk in Tris-buffered saline with Tween 20 (TBST; 10 mM Tris–HCl, pH 8.0; 150 mM NaCl; and 0.5% Tween 20) for 2 h. Next, after being washed with TBST three times, the membrane was incubated with primary antibodies (Table S2) overnight at 4 °C. After being washed with TBST three times, horseradish peroxidase-conjugated secondary antibody diluted in non-fat dry milk (diluted 1:5000) was added and incubated at room temperature for 1 h. The membrane was removed from boxes with blunt forceps after being washed with TBST at least three times. The protein expression of each indicated group was detected with an enhanced chemiluminescence system (Tanon-4800, Shanghai, China). The expression of the cytoplasmic protein was normalized to β-actin or tubulin using ImageJ software.

### Histological staining

Samples originating from humans and rat intervertebral disc tissues were dissected and fixed in 4% paraformaldehyde. After decalcification in 10% EDTA, the samples were processed, embedded in paraffin, and cut into 5-μm sections using a microtome. Standard hematoxylin and eosin staining, Safranin O and fast green staining, Masson staining, and Alcian staining were performed according to the manufacturer's recommended procedure.

### Immunohistochemistry

Murine skin tissues from two groups were cut into 5-μm-thick sections. After gradient alcohol dewaxing and antigen repairing, these slices were blocked in goat serum at room temperature for 30 min and then incubated with anti-iNOS (diluted 1:200, 20886-1-AP, Proteintech, USA) and anti-COX2 (diluted 1:200, #2859, Cell Signaling Technology, USA) antibodies at 4 °C overnight, followed by incubation with a horseradish peroxidase-conjugated secondary antibody at room temperature for 60 min. Detection was performed using the VECTASTAIN Elite ABC kit (Vector, Burlingame, CA, USA), and incubation with 0.5 mg/mL 3,3′-diaminobenzidine in 50 mM Tris-Cl (Sigma Aldrich) was performed for visualization. Then, the slides were counterstained with 1% hematoxylin.

### Immunofluorescence staining

Immunofluorescence staining of rat NP cells was performed with anti-PTX3 (diluted 1:200, 20886-1-AP, Proteintech, USA) antibodies. The procedure was conducted as described previously, and images were taken with a fluorescence microscope (Nikon, Japan).

### Cell viability

Cell viability was performed using CCK-8 assay (A311, Vazyme, Nanjing, China) following the manufacturer's protocols. Rat NP cells were seeded into 96-well plates at a density of 5 × 10^3^ per well and treated with associated stimulation. After being washed with PBS three times, cells of each group were added with 100 μL culture media containing 10% CCK-8 solution and then incubated in the dark for 2 h. The absorbance at a wavelength of 450 nm was determined with a microplate reader.

### Transmission electron microscopy

As previously reported, all cells were collected by trypsinization, transferred into 2 mL centrifuge tubes, and fixed with fixation solution (Servicebio, G1102) at 4 °C for 2 h. The cells were post-fixed in 1% osmium tetroxide in 0.1 M phosphate buffer (pH 7.4) at room temperature for 2 h. Afterward, the NP cells were dehydrated in a graded ethanol series (50%, 70%, 80%, 90%, 95%, 100%, 100%) for 15 min for each solution and infiltrated with propylene oxide to embedding medium overnight. Ultrathin sections (50 nm) were obtained using an EMUC7 ultramicrotome (Leica), post-stained with uranylacetate and lead citrate, and visualized using a transmission electron microscope (HT7700; Hitachi, Tokyo, Japan).

### JC-1 assay

JC-1 assay kit (C2003S, Beyotime Biotechnology, China) was used to detect the mitochondrial membrane potential. According to the manufacturer's instructions, rat NP cells from each indicated group in 24-well plates were stained with a JC-1 staining solution at 37 °C for 20 min while protected from light. Then, each well in the plate was washed twice with 1 × JC-1 staining buffer, and the fluorescence intensity was measured with a fluorescence microscope (Nikon, Japan).

### Calcein AM-PI staining

Rat NP cells were seeded into a 24-well plate at a density of 2 × 10^4^ per well. The cells in each group were treated with relevant stimulations and then washed with PBS for one time. According to the manufacturer's instructions for the Calcein AM-PI staining kit (C2015, Beyotime Biotechnology, China), each well of a 24-well plate was added with 250 μL buffer containing 0.25 μL calcein AM (1000 ×) and 0.25 μL propidium iodide (PI) (1000 ×). Then the plate was incubated at 37 °C for 30 min and detected with a fluorescence microscope (Nikon, Japan).

### Caspase 3 activation detection

Caspase 3 activation was detected using a commercial GreenNuc™ Live Cell Caspase-3 Activity Detection Kit (C1168, Beyotime Biotechnology, China). According to the manufacturer's introductions, cells of each indicated group after relevant stimulation were incubated with 5 μM GreenNuc™ Caspase-3 substrate and protected from light for 30 min. Activation of caspase 3 could be visible in green fluorescence with a fluorescence microscope (Nikon, Japan).

### Flow cytometry

The apoptotic rate of each group was performed by an Annexin V-FITC/PI Apoptosis Detection Kit (A211, Vazyme, Nanjing, China). According to the manufacturer's instructions, adherent rat NP cells were suspended by 0.25% trypsin (HyClone, Logan, USA) in a 100 μL volume of 1 × binding buffer. Then 5 μL Annexin V-FITC dye and 5 μL PI were added separately. Then it was incubated in the dark at room temperature for 15 min. In the end, 200 μL 1 × binding buffer was added into the tube and then assayed on a FACS Calibur flow cytometer (BD Biosciences, USA). The data obtained from this assay were analyzed with FlowJo v10 software (USA).

### MRI

A 3.0 T MRI scanner (GE Signa HDX, USA) was used to scan the images of the rat tail. The structure of NP was visible and the area was measured on a T2-weighted sequence. Especially, the tails of rats were straightened.

### X-ray

The same needle-punctured segmental intervertebral disc was performed with an X-ray (GE XR650, USA). Digital images were obtained using the radiographic plate system. The disc height index was measured by Image J. In this study, we proposed a modified measuring method of calculating the disc height index. Briefly, only six (A, B, C, D, E and F) numerical values were measured to largely reduce errors.

### Statistical analysis

All statistical analyses were performed using GraphPad Prism 8.0.1 (USA). Data were presented as mean ± standard deviation. Both *t*-test and one-way ANOVA were used to analyze the data. For ANOVA, Bonferroni post hoc analysis was used to compare multiple groups. Statistical significance was indicated when *p* < 0.05.

## Results

### FSP1 is up-regulated during IDD and responsive to TNFα

**Figure 1:**
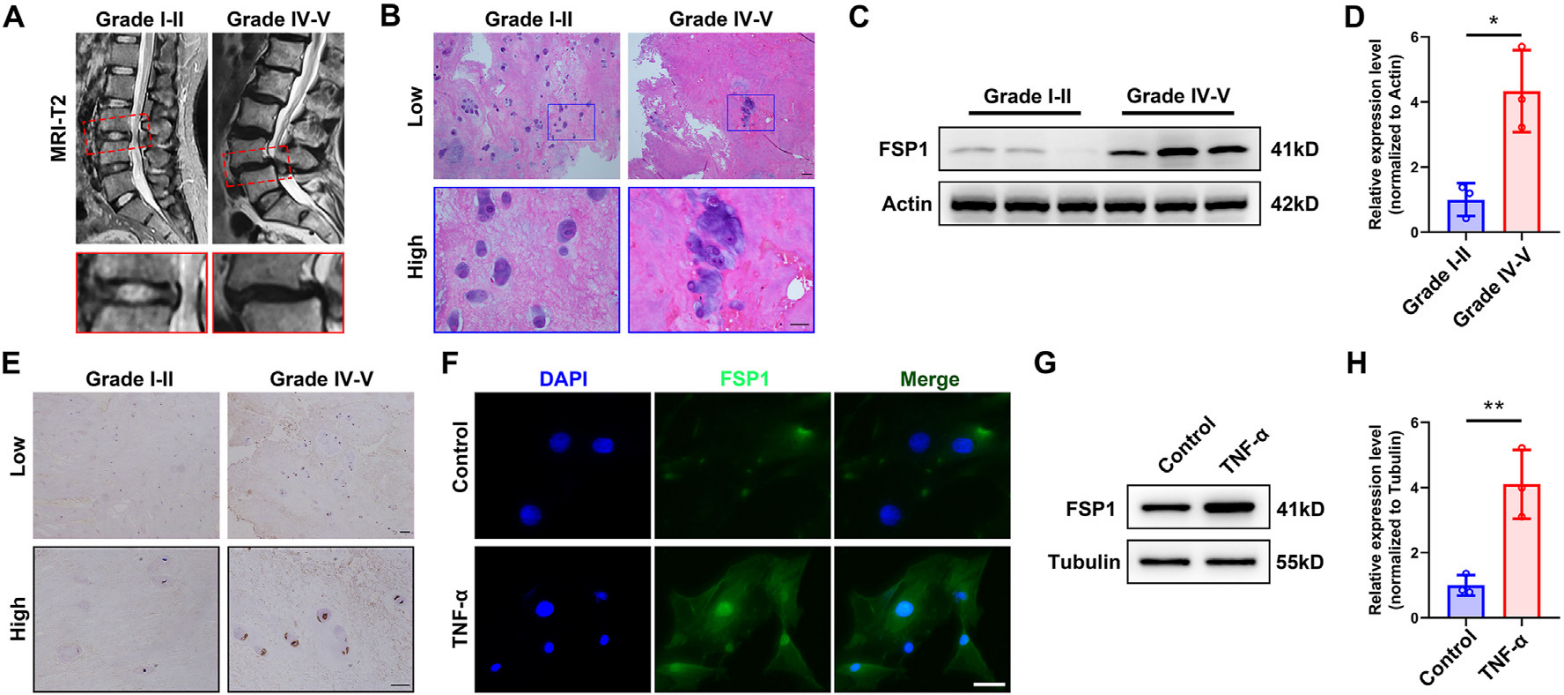
FSP1 is up-regulated during IDD and responsive to TNFα. **(A)** Representative MRI-T2 images of the lumbar spine from IDD patients with Pfirrmann grade II or grade V respectively. The lower panels show pictures of the indicated segment at a high magnification. **(B)** Hematoxylin and eosin **(H&E**) staining on human NP tissue in two groups. Scale bar, 100 μm. **(C, D)** Western blot of FSP1 expression in two groups and its quantification. **(E)** Immunohistochemistry staining of FSP1 in two groups. Scale bar, 100 μm. **(F)** Immunofluorescence staining of FSP1 in rat NP cells with or without TNFα stimulation. Scale bar, 20 μm. **(G, H)** Western blot of FSP1 expression in rat NP cells with or without TNFα stimulation and its quantification. FSP1, ferroptosis suppressor protein 1; IDD, intervertebral disc degeneration; TNFα, tumor necrosis factor alpha; NP, nucleus pulposus. ∗*P* < 0.05, ∗∗*P* < 0.01.

To determine the expression level of FSP1 in the control (Grade I–II) group and degenerative (Grade IV–V) group, 25 patients were included in this study (Table S1). Based on the Pirrmann grading system, there were 7 patients in the control group and 18 patients in the degenerative group as validated by MRI in T2WI (Fig. 1A). Total intervertebral disc tissues were obtained after spinal surgery. Then histological staining of NP such as hematoxylin and eosin staining was performed (Fig. 1B). Compared with the control group, FSP1 levels were significantly increased in the human NP tissue of the degenerative group (Fig. 1C, D). As confirmed by immunohistochemistry staining of FSP1 (Fig. 1E), higher expression of FSP1 was observed in NP cells of the degenerative group. However, FSP1 was mostly elevated in cytoplasm localization as presented in the magnified panel. To mimic the degenerative occurrence *in vitro*, the established pro-inflammatory cytokine TNFα was utilized in the stimulation of rat NP cells. Thereafter, as identified by both immunofluorescence staining (Fig. 1F) and Western blot (Fig. 1G, H), TNFα dramatically enhanced the expression of FSP1 in rat NP cells. The results mentioned above uncover that FSP1 is up-regulated in degenerative NP tissues and responsive to TNFα, suggesting that FSP1 is involved in IDD.

### Inhibition of FSP1 fails to induce ferroptosis

**Figure 2:**
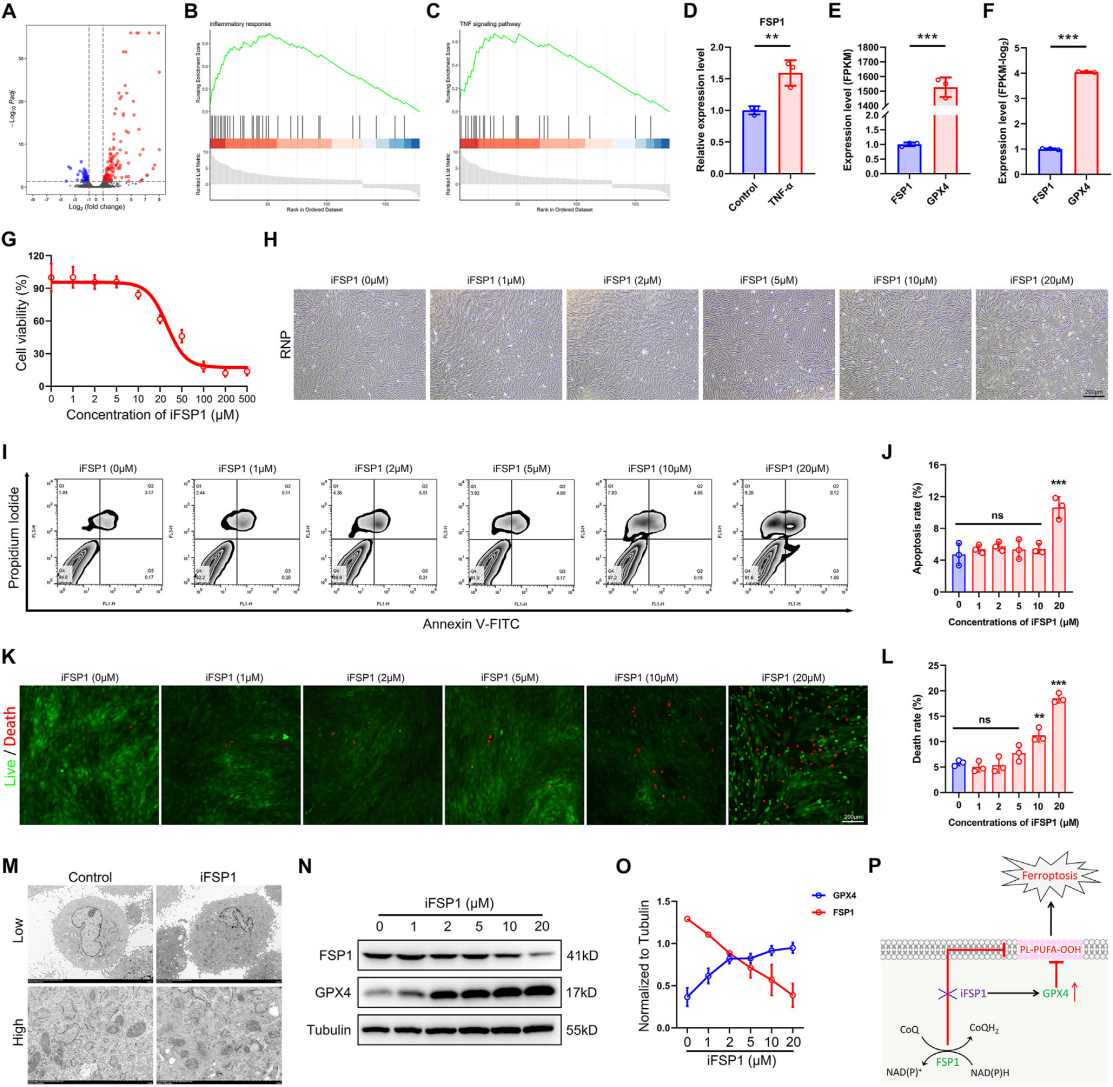
Inhibition of FSP1 fails to induce ferroptosis. **(A**–**C)** Rat NP cells were treated with or without TNFα and RNA sequencing was performed. The differentially expressed genes were presented in the volcano plot. Inflammatory response and TNF signaling pathway were activated on GSEA. **(D)** FSP1 was up-regulated after TNFα stimulation. **(E, F)** Transcriptional levels of FSP1 and GPX4 in normal NP cells. **(G)** Cell viability of NP cells with different concentrations of iFSP1 treatment. **(H)** Cell morphology after treatment with different doses of iFSP1 (0, 1, 2, 5, 10, and 20 μM). Scale bar, 200 μm. **(I, J)** Cell apoptosis of NP cells after treatment with different concentrations of iFSP1 (0, 1, 2, 5, 10, and 20 μM) and quantification of apoptotic cells was performed by flow cytometry. **(K, L)** Live-death staining in NP cells with stimulation by different doses of iFSP1 and death rate (%) calculation. Scale bar, 200 μm. **(M)** Cell morphology of transmission electron microscope in control and iFSP1 treated groups. Low, 5 μm; high, 1 μm. **(N, O)** Western blot of FSP1 and GPX4 expression in rat NP cells with stimulation by different doses of iFSP1 and their quantification. **(P)** Schematic diagram of the role of FSP1 and GPX4 in fighting against ferroptosis in NP cells. FSP1, ferroptosis suppressor protein 1; NP, nucleus pulposus; TNFα, tumor necrosis factor alpha; GPX4, glutathione peroxidase 4. ∗∗*P* < 0.01, ∗∗∗*P* < 0.001.

According to previous studies, FSP1 is a critical gatekeeper to fight against ferroptosis. With the stimulation by TNFα and verification by RNA sequencing, a series of inflammatory cytokines and chemokines were up-regulated (Fig. 2A), and the inflammatory process and TNF signaling pathway were activated (Fig. 2B, C). Herein, FSP1 was identified to be enhanced by TNFα inducement (Fig. 2D). However, the first-line anti-ferroptosis protein GPX4 was discovered to dramatically express compared with FSP1 (Fig. 2E, F). The following experiments aimed to identify whether FSP1 inhibition alone could trigger ferroptosis in rat NP cells. The innovative FSP1 inhibitor, iFSP1, was a small molecule used in this study. With the iFSP1 concentrations increasing, obvious rat NP cell death appeared at 20 μM iFSP1 in cell viability (Fig. 2G). Further flow cytometry implied a prominent cell death with 20 μM iFSP1 stimulation (Fig. 2H–J). This observation was also identified by live-death staining (Fig. 2K, L). However, there was no obvious ferroptotic phenotype after 20 μM iFSP1 stimulation under a transmission electron microscope (Fig. 2M). In terms of the key role of FSP1 and GPX4 in anti-ferroptosis, the expression of ferroptotic proteins (GPX4, FSP1, ACSL4, Nrf2, and SLC7A11) was examined and quantified (Fig. 2N, O; Fig. S1), whereupon it seems the expression of GPX4 and FSP1 is in reverse. Overall, GPX4 may exhibit a compensatory role in FSP1 deprivation to fight against ferroptosis (Fig. 2P).

### FSP1-evoked apoptosis by TNFα is related to caspase 3 activation and mitochondrial damage

**Figure 3:**
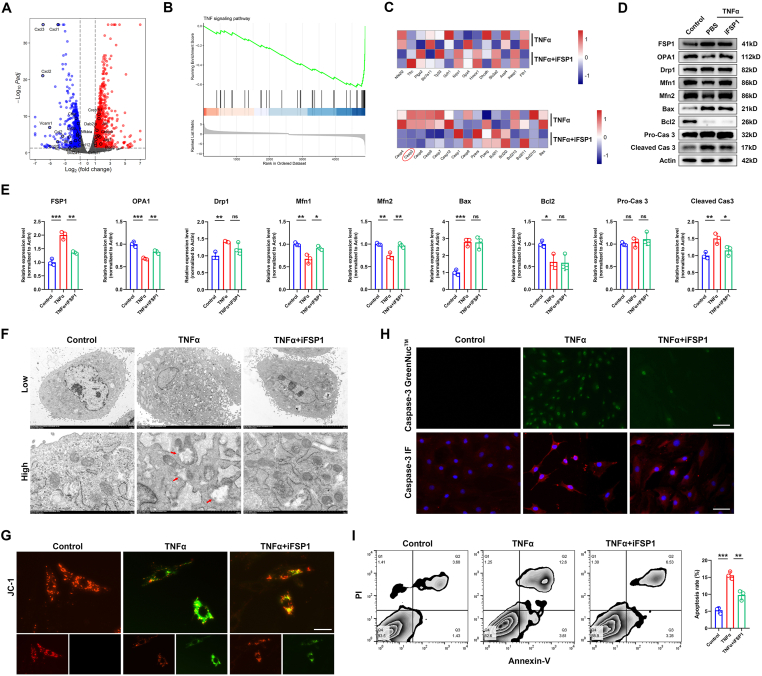
FSP1-evoked apoptosis by TNFα is related to caspase 3 activation and mitochondrial damage. **(A, B)** TNFα stimulated rat NP cells were co-cultured with or without iFSP1 and RNA sequencing was performed. The differentially expressed genes were presented in the volcano plot and the TNF signaling pathway was inhibited on GSEA after iFSP1 treatment. **(C)** The heat map revealed no significance of ferroptotic genes between the two groups, while apoptotic genes containing caspase 3 and caspase 4 were both down-regulated. **(D, E)** Western blot analysis of FSP1, OPA1, Drp1, Mfn1, Mfn2, Bax, Bcl2, pro-caspase 3, and cleaved caspase 3 in TNFα stimulated rat NP cells with or without iFSP1 treatment and their quantification. **(F)** Transmission electron microscope of TNFα stimulated rat NP cells with or without iFSP1 treatment. Low, 5 μm; high, 1 μm. **(G)** JC-1 staining of NP cells of indicated three groups. Scale bar, 20 μm. **(H)** Caspase-3 GreenNuc™ staining and caspase-3 immunofluorescence in TNFα stimulated rat NP cells with or without iFSP1 treatment. Scale bar, 100 μm. **(I)** Annexin V/PI staining of three groups and apoptotic rate (%) quantification. FSP1, ferroptosis suppressor protein 1; NP, nucleus pulposus; PI, propidium iodide; TNFα, tumor necrosis factor alpha; ns, not significance. ∗*P* < 0.05, ∗∗*P* < 0.01, ∗∗∗*P* < 0.001.

To detect whether FSP1 inhibition could impair TNFα-mediated inflammatory pathway activation, the FSP1 inhibitor iFSP1 (5 μM) was added with or without TNFα stimulation (Fig. 3A). As a result, the TNF signaling pathway was significantly inhibited (Fig. 3B). Moreover, the expression of both ferroptotic (*Nfe2l2*, *Trfc*, *Ptgs2*, *Slc7a11*, *Tp53*, *Gch1*, *Nqo1*, *Gpx4*, *Hmox1*, *Dhodh*, *Slc3a2*, *Acsl4*, *Keap1*, and *Fth1*) and apoptotic (*Casp4*, *Casp3*, *Casp8*, *Casp6*, *Casp7*, *Casp12*, *Casp1*, *Casp9*, *Ppara*, *Pparg*, *Bcl2l1*, *Bcl2l2*, *Bcl2l13*, *Bcl2l11*, *Bcl2l10*, and *Bax*) genes was analyzed, but there was no significant difference in ferroptosis markers while *Casp3* and *Casp4* were down-regulated after iFSP1 treatment (Fig. 3C). To test whether mitochondria and apoptosis-related proteins involve in iFSP1-reliant TNFα signaling suppression, FSP1 and associated markers (OPA1, Drp1, Mfn1, Mfn2, Bax, Bcl2, pro-caspase 3, and cleaved caspase 3) were examined and quantified by Western blot (Fig. 3D, E). In this part, iFSP1 treatment dramatically reversed the TNFα-induced high expression of Drp1 and low expression of OPA1, Mfn1, and Mfn2, and further repressed the activation of cleaved caspase 3, while presenting no significant effect on the expression of Drp1, Bcl2, and Bax. In addition, iFSP1 treatment with TNFα stimulation obviously displayed mild mitochondrial damages compared with TNFα treatment alone (Fig. 3F, G). The activation of caspase 3 is critical for cell apoptosis. As determined by caspase-3 GreenNuc™ and caspase-3 immunofluorescence, iFSP1 dramatically reversed TNFα-mediated caspase 3 activation (Fig. 3H). Ultimately, iFSP1 also debilitated TNFα-mediated rat NP cell apoptosis (Fig. 3I).

### *Fsp1* knockdown reduces cellular reactivity to TNFα and dampens the apoptotic phenotype

**Figure 4:**
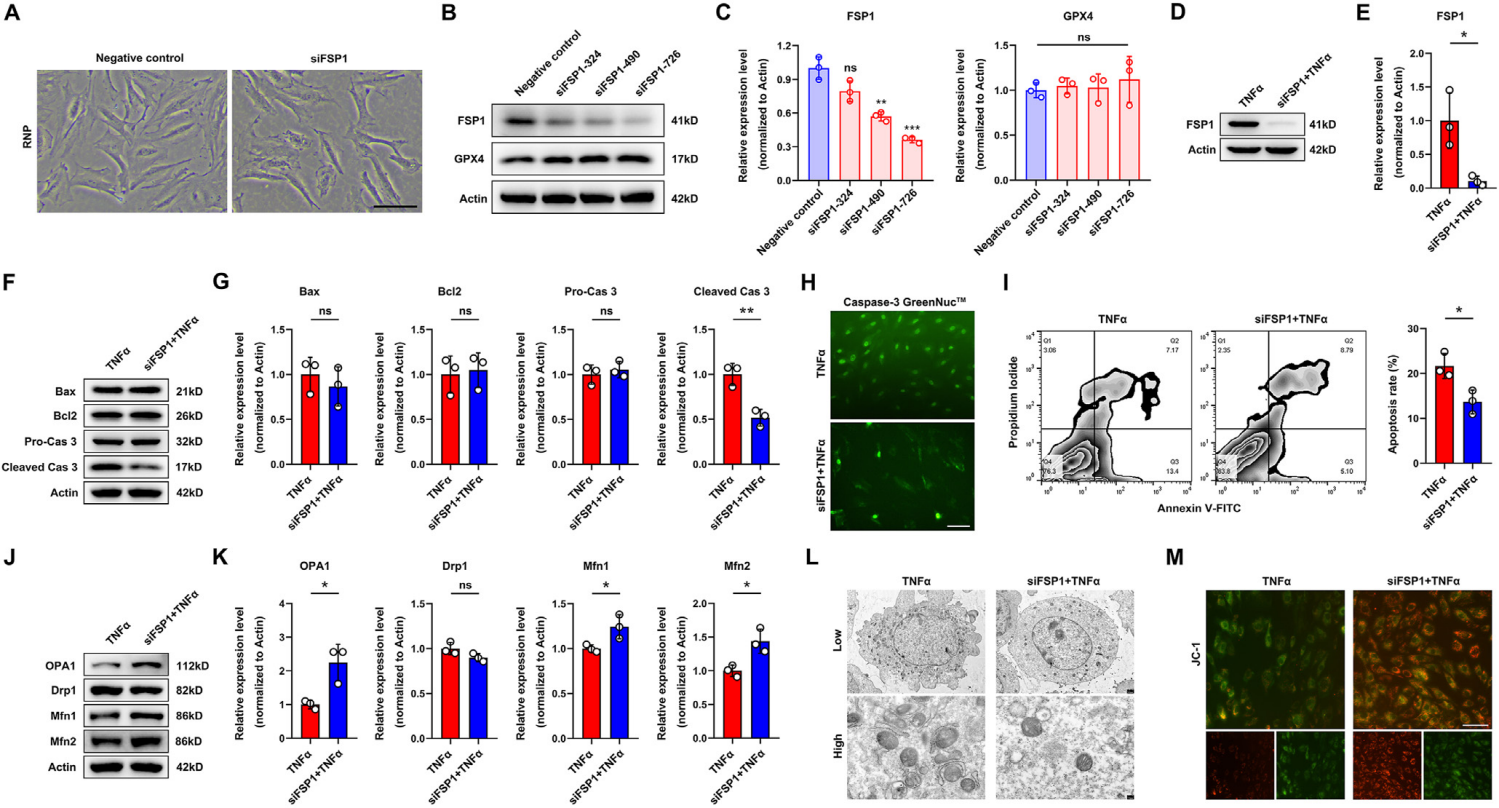
*Fsp1* knockdown reduces cellular reactivity to TNFα and dampens the apoptotic phenotype. **(A)** Cell morphology in normal NP cells or after siFSP1 transfection. **(B, C)** Western blot analysis of FSP1 and GPX4 after three times of siFSP1 transfection, and their quantification. **(D, E)** Western blot analysis of FSP1 in TNFα stimulated NP cells with or without siFSP1 transfection, and its quantification. **(F, G)** Western blot analysis of Bax, Bcl2, pro-caspase 3, and cleaved caspase 3 in TNFα stimulated NP cells with or without siFSP1 transfection, and their quantification. **(H)** Caspase-3 GreenNuc™ staining in TNFα stimulated rat NP cells with or without siFSP1 transfection. Scale bar, 100 μm. **(I)** Annexin V/PI staining in TNFα stimulated rat NP cells with or without siFSP1 transfection, and apoptotic rate (%) quantification. **(J, K)** Western blot analysis of OPA1, Drp1, Mfn1, and Mfn2 in TNFα stimulated NP cells with or without siFSP1 transfection, and their quantification. **(L)** Transmission electron microscope of TNFα stimulated rat NP cells with or without siFSP1 transfection. Low, 5 μm; high, 1 μm. **(M)** JC-1 staining of NP cells of indicated two groups. Scale bar, 20 μm. FSP1, ferroptosis suppressor protein 1; PI, propidium iodide; TNFα, tumor necrosis factor alpha; GPX4, glutathione peroxidase 4; ns, not significance. ∗*P* < 0.05, ∗∗*P* < 0.01, ∗∗∗*P* < 0.001.

To determine *Fsp1* gene in TNFα-mediated apoptosis, small interfering RNAs were designed to decline the expression of FSP1. After small interfering RNA transfection (siFSP1-324, siFSP1-490, and siFSP1-726), cell morphology was discovered with no difference compared with controls (Fig. 4A). The efficacy of FSP1 inhibition dramatically appeared in siFSP1-726 as detected by Western blot (Fig. 4B, C), while the expression of GPX4 was invariable. Herein, the expression of endogenous FSP1 was significantly inhibited by siFSP1 when stimulated with TNFα (Fig. 4D, E). Apoptotic markers (Bax, Bcl2, pro-caspase 3, and cleaved caspase 3) in two TNFα stimulation groups with or without treatment of siFSP1 were detected by Western blot, and the results revealed that FSP1 inhibition remarkably decreased the activation of caspase 3 while was irrelevant to Bcl2/Bax axis (Fig. 4F, G). This finding was also demonstrated by the direct determination of active caspase 3 in caspase-3 GreenNuc™ (Fig. 4H). Accordingly, as examined by flow cytometry, TNFα-reliant cell death was diminished with FSP1 inhibition (Fig. 4I). Besides, mitochondria-related proteins (OPA1, Drp1, Mfn1, and Mfn2) were detected by Western blot and the results verified that siFSP1 reversed TNFα-mediated mitochondrial damage (Fig. 4J, K) and that this effect could be directly observed by transmission electron microscope (Fig. 4L). Ultimately, endogenous FSP1 inhibition by siFSP1 effectively sustained the mitochondrial membrane potential as shown by JC-1 staining (Fig. 4M).

### Exogenous FSP1 is incapable of triggering cell death and unable to potentiate TNFα-derived cell demise

**Figure 5:**
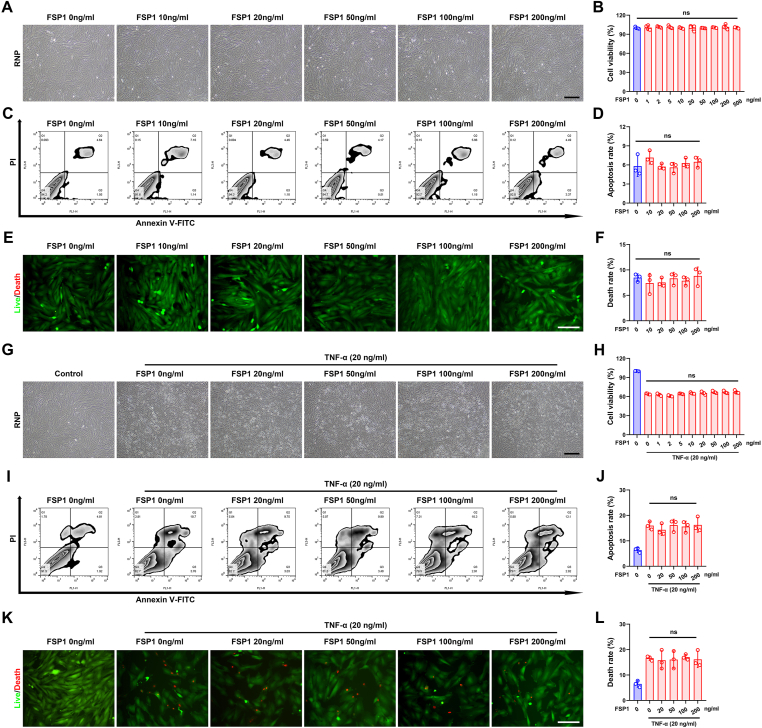
Exogenous FSP1 is incapable of triggering cell death and unable to potentiate TNFα-derived cell demise. **(A)** Cell morphology of NP cells after treatment of different recombinant FSP1 concentrations (0, 10, 20, 50, 100, and 200 ng/mL). Scale bar, 200 μm. **(B)** Cell viability of NP cells after treatment of different recombinant FSP1 concentrations. **(C, D)** Annexin V/PI staining in rat NP cells with addition of different doses of FSP1, and apoptotic rate (%) quantification. **(E, F)** Live-death staining in NP cells with stimulation by different doses of FSP1, as well as death rate (%) calculation. Scale bar, 100 μm. **(G)** Cell morphology of TNFα (20 ng/mL) stimulated NP cells after treatment of different recombinant FSP1 concentrations (0, 20, 50, 100, and 200 ng/mL). Scale bar, 200 μm. **(H)** Cell viability of TNFα stimulated NP cells after treatment of different recombinant FSP1 concentrations. **(I, J)** Annexin V/PI staining in TNFα stimulated rat NP cells with addition of different doses of FSP1, and apoptotic rate (%) quantification. **(K, L)** Live-death staining of TNFα stimulated NP cells with stimulation by different doses of FSP1 and death rate (%) calculation. Scale bar, 100 μm. FSP1, ferroptosis suppressor protein 1; PI, propidium iodide; TNFα, tumor necrosis factor alpha; ns, not significance.

To identify whether exogenous recombinant FSP1 triggers rat NP cell death, these cells were treated with a dose gradient of recombinant FSP1. The cell morphologies were unchanged when co-cultured with 0–200 ng/mL FSP1 (Fig. 5A). Moreover, differences in cell viability (Fig. 5B) and cell death rate (Fig. 5C, D), as well as live-death staining (Fig. 5E, F), were insignificant in different concentration groups. As TNFα is corroborated for the inducer of FSP1 and exaggerates IDD, both TNFα (20 ng/mL) and different exogenous recombinant FSP1 concentrations were co-added to rat NP cells. The results delineated that TNFα remarkably exacerbates rat NP cells' lower viability while FSP1 does not present synergism to this condition (Fig. 5G, H). Additionally, recombinant FSP1 showed no impact on TNFα-mediated rat NP cell death, as detected by flow cytometry (Fig. 5I, J) and live-death staining (Fig. 5K, L).

### Inhibition of FSP1 ameliorates IDD in rats

**Figure 6:**
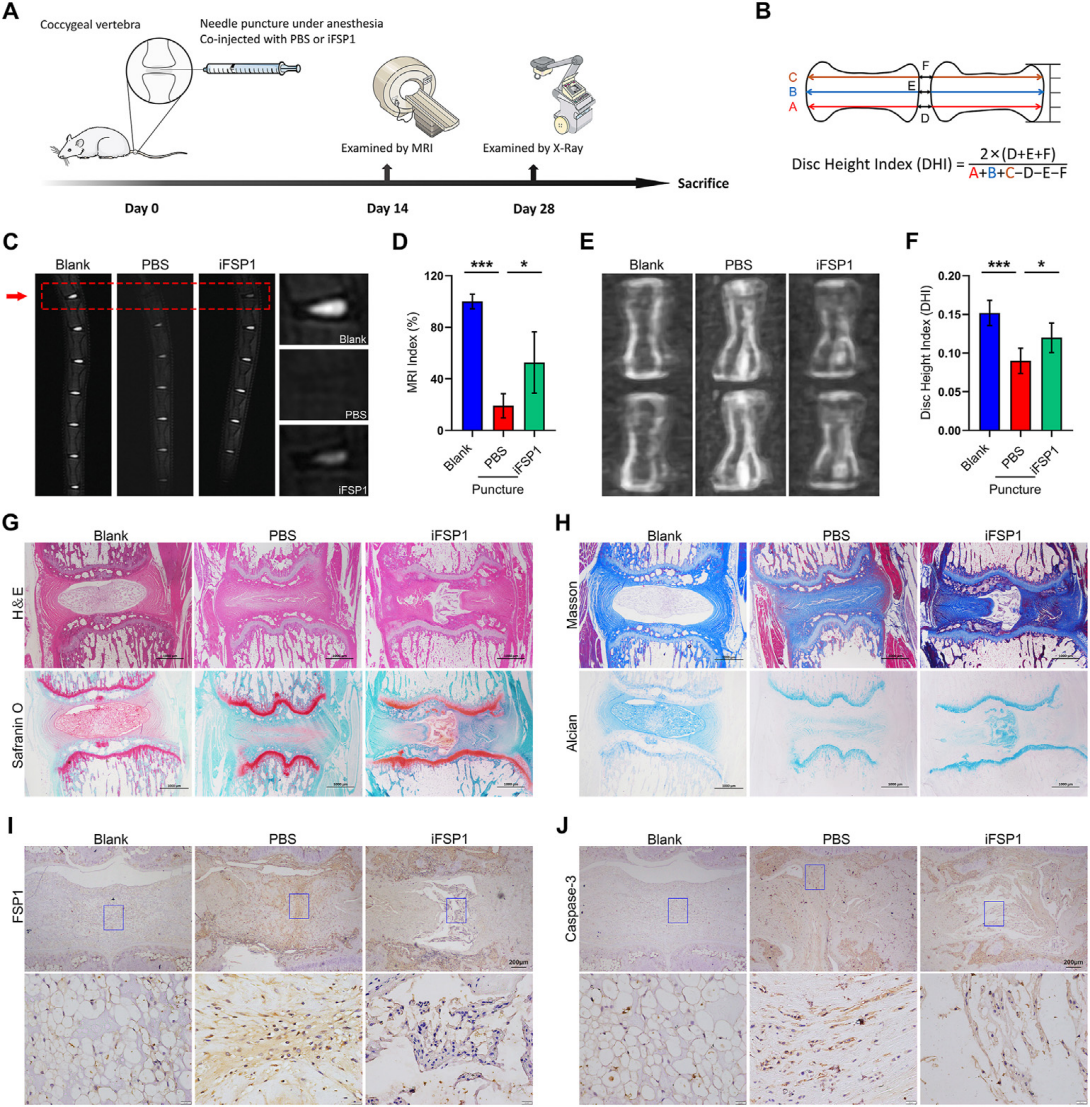
Inhibition of FSP1 ameliorates intervertebral disc degeneration in rats. **(A)** A needle puncture rat model was established and radiological tests were performed on time-line. **(B)** A sketch map of the novel method for calculation of DHI. **(C, D)** MRI of rat tails on three indicated groups and MRI index (%) calculation by ImageJ. **(E, F)** X-ray of rat tails of three indicated groups and DHI calculation. **(G, H)** Histological investigations comprising hematoxylin and eosin **(H&E**) staining, Safranin O staining, Masson staining, and Alcian Blue staining were performed in three groups. Scale bar, 1000 μm. **(I, J)** Immunohistochemistry of FSP1 and caspase 3 of three groups. Scale bar, 200 μm in low magnification and 20 μm in high magnification. FSP1, ferroptosis suppressor protein 1; MRI, magnetic resonance imaging; DHI, disc height index. ∗*P* < 0.05, ∗∗∗*P* < 0.001.

Further studies were projected to elucidate the role of FSP1 inhibition in IDD, and thereunder a well-known needle puncture rat model was established (Fig. 6A) and radiological tests were located on a timeline. A novel method of the calculation of the disc height index is presented in Figure 6B. Compared with the PBS group, the iFSP1 group had a higher MRI index (Fig. 6C, D) on disc signal brightness and a better disc height index from the X-ray (Fig. 6E, F). These discs from each indicated group were subjected to histological staining. The results documented that the iFSP1 group after needle puncture retained disc shape and prevented the degradation of the cartilaginous matrix compared with the PBS group (Fig. 6G). Meanwhile, Masson staining and Alcian Blue staining both revealed collagenous reconstruction and mild proteoglycan loss in the iFSP1 group (Fig. 6H). To specifically identify molecular biomarker expression in each indicated group, immunohistochemistry was implemented. The results manifested that iFSP1 reversed needle puncture-induced high expression of both FSP1 and caspase 3 (Fig. 6I, J). Overall, these data suggest that FSP1 inhibition ameliorates IDD *in vivo*.

### FSP1 is downstream of TNFα and mediates NF-κB signaling activation

**Figure 7:**
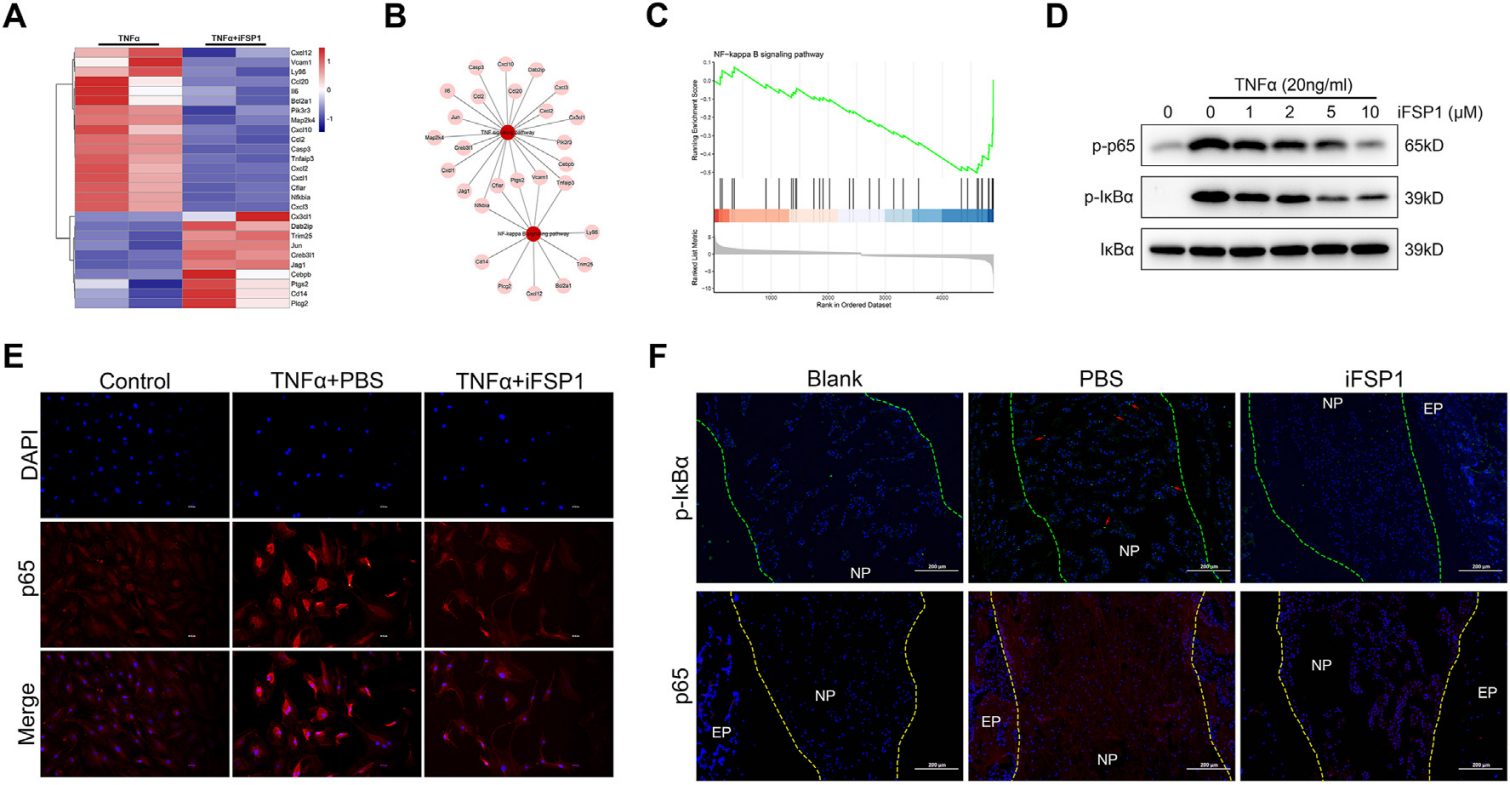
FSP1 is a downstream of TNFα and mediates NF-κB signaling activation. **(A)** TNFα stimulated rat NP cells were co-cultured with or without iFSP1 and RNA sequencing was performed. The differentially expressed genes of NF-κB signaling were shown in heat maps. **(B)** Independent or conjoint genes in TNF signaling and NF-κB signaling. **(C)** NF-κB signaling pathway was inhibited on GSEA after iFSP1 treatment. **(D)** Western blot of p-p65 and p-IκBα in TNFα stimulated NP cells after treatment of different iFSP1 concentrations. **(E)** Immunofluorescence staining of p65 in TNFα stimulated NP cells in the existence or absence of iFSP1. Scale bar, 29.34 μm. **(F)** Immunofluorescence staining of p65 and p-IκBα in rat intervertebral disc tissues of three groups. Scale bar, 200 μm. FSP1, ferroptosis suppressor protein 1; TNFα, tumor necrosis factor alpha.

**Figure 8:**
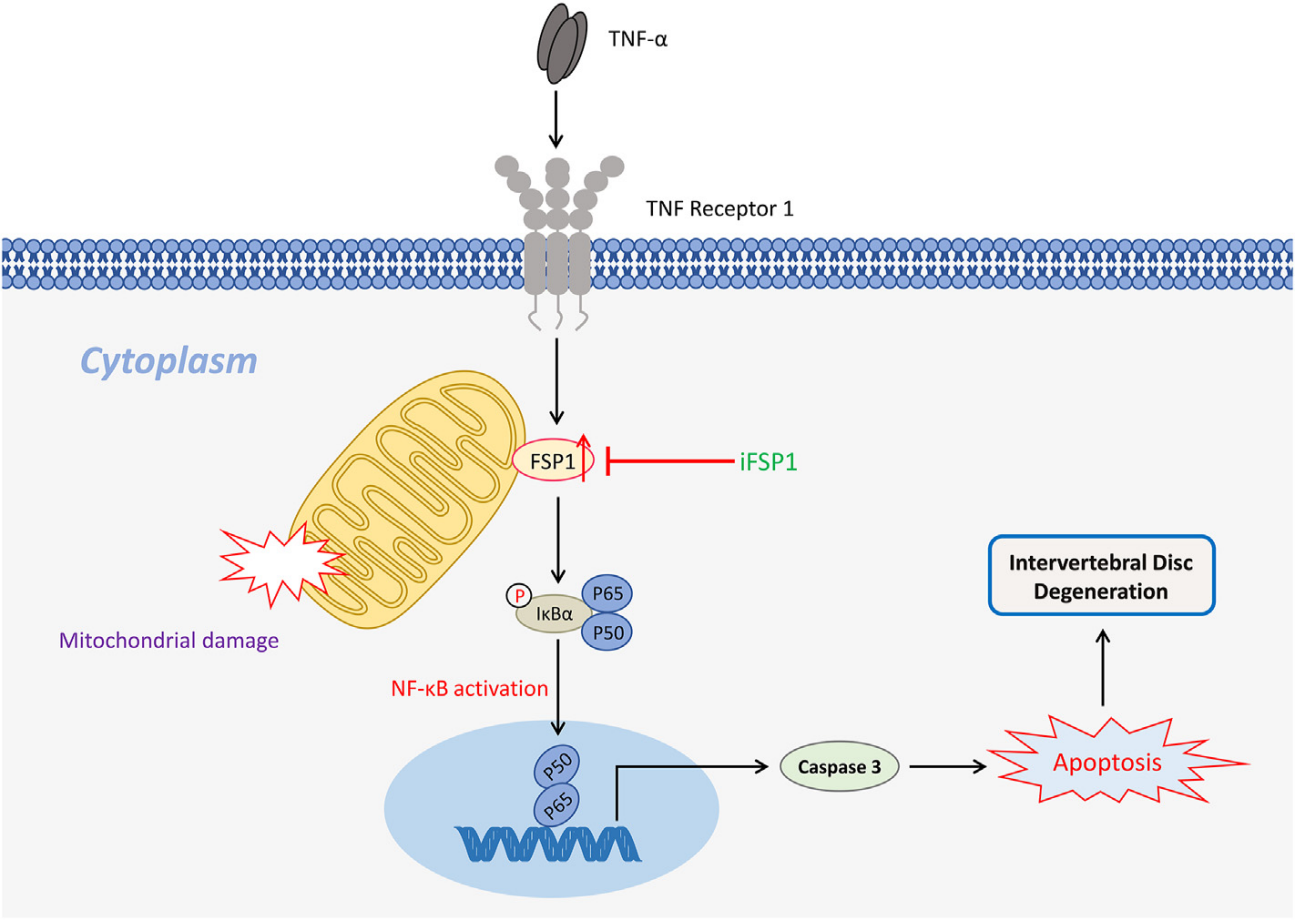
The schematic depicting a proposed model for the function of ferroptosis suppressor protein 1 (FSP1) in intervertebral disc degeneration.

To figure out the downstream signaling mechanisms of TNFα-mediated up-regulation of FSP1 that could accelerate IDD, RNA sequencing in TNFα-treated groups in the existence or absence of iFSP1 was performed. These data corroborated that NF-κB signaling was significantly inhibited by iFSP1 with the stimulation of TNFα (Fig. 7A–C). Moreover, the expression of p-p65 and p-IκBα in rat NP cellular cytoplasm was dose-dependent on iFSP1 with TNFα stimulation (Fig. 7D). In nuclei, iFSP1 treatment dramatically inhibited nuclear translocation of p65 (Fig. 7E). Similarly, iFSP1 significantly diminished the expression of p65 and p-IκBα *in vivo* (Fig. 7F). Taken together, these above demonstrate that FSP1 is downstream of TNFα and mediates NF-κB signaling activation to promote IDD (Fig. 8).

## Discussion

IDD is a common degenerative causative factor for lower back pain. Herein, this study demonstrates the ferroptosis defense protein FSP1 is critical for the promotion of IDD. Moreover, FSP1 is downstream of TNFα and mediates NF-κB signaling activation, thus resulting in caspase 3 activation and mitochondrial damage. Overall, FSP1 inhibition might be regarded as a promising therapeutic strategy to attenuate TNFα-reliant inflammatory process and ameliorate IDD.

IDD is commonly associated with chronic inflammation.^17^^,^^45^ Processive immune infiltration and inflammatory cytokine secretion alter the microenvironment in the disc, whereupon the proinflammatory cytokine TNFα plays a key role during IDD.^11^ TNFα accelerates the degradation of the extracellular matrix and triggers NP cell death since anti-TNFα approaches draw feasible propensity for the prevention of IDD.^14^^,^^46^ A previous reported proapoptotic protein, AIFM2, also known as FSP1, is a nuclear factor that possesses DNA binding activity to facilitate cell death.^34^^,^^38^^,^^47^ Intriguingly, FSP1 was up-regulated in degenerative NP tissues and in line with TNFα stimulation in NP cells. It is known that various cell death types containing apoptosis and ferroptosis are both involved in IDD. The up-regulation of FSP1 raises the interest to figure out how FSP1 functions during this process.

Currently, multifarious forms of cell death are occurring during the development of IDD.^19^, ^20^, ^21^, ^22^, ^23^, ^24^ Ferroptosis is a newly identified iron-dependent and non-apoptotic programmed cell death that is related to lipid peroxidation.^48^, ^49^, ^50^ FSP1 is corroborated as an endogenous anti-ferroptosis protein to confer the induction by ferroptosis inducers when lacking GPX4.^35^^,^^36^ However, previous studies documented FSP1 as a pro-apoptosis protein with high expression that could induce cell death under apoptotic agent treatment.^51^^,^^52^ Therefore, the relationships between ferroptosis and apoptosis are required to be uncovered, and the main attention is paid to whether there is a bidirectional molecule serving as the watershed in two forms of cell death.^53^^,^^54^ In NP cells, the expression of GPX4 is much higher than FSP1 so NP cells are a GPX4-dominant ferroptosis gatekeeper cell line. The ferroptosis tendency in NP cells seems elevated after iFSP1 stimulation as compensatory GPX4 upregulation and FSP1 inhibition. Although glutathione and coenzyme Q_10_ are respectively responsible for GPX4 and FSP1, NADPH is vital for both two reduction processes.^36^^,^^55^ Accordingly, GPX4 is more important than FSP1 for NP cells to fight against ferroptosis.

Previous studies have already revealed that FSP1 induces apoptosis in a caspase-independent and p53-independent manner.^34^^,^^37^ FSP1, also known as AIFM2 or AMID, is an AIF-homologous member that encodes a 373 amino acid protein while lacking recognizable mitochondrial localization sequence to access inner mitochondria that forms a ring-like shape around mitochondria.^40^ When FSP1 overexpresses, cell apoptosis appears following a dose-dependent manner.^34^ In this study, FSP1 is induced by proinflammatory cytokine TNFα thus mediating caspase 3 activation and mitochondria damage. From the localization of FSP1, it may be speculated that TNFα-induced high FSP1 expression affects the stability of the mitochondrial membrane, leading to alteration of mitochondrial potential. Notwithstanding, TNFα-induced cell apoptosis is caspase-reliant, so high FSP1 expression is in line with caspase 3 activation.^14^^,^^56^ This observation does not contradict previous findings as TNFα sensitizes NF-κB signaling activation through FSP1 and then facilitates caspase 3 transcription. Antecedent investigations discovered that FSP1 acts as a transportation factor and enters into the nucleus to trigger downstream gene activation directly. In the context of IDD, FSP1 might drive cell apoptosis by increasing DNA binding activity.

As mentioned before, NP cells are GPX4-dominant to counteract ferroptosis. FSP1 inhibition alone by target disruption fails to provoke ferroptotic phenotype in NP cells. At the same time, the inhibition role of iFSP1 for FSP1 mitigates NP cell apoptosis and alleviates IDD. *In vivo* experiments indicate iFSP1 acts on alleviation of NP tissue degeneration by *in situ* injection instead of oral or intravenous administration. In clinic, it is better to avoid invasive treatment so that more inhibitors targeting FSP1 could give optimal prospects. Nevertheless, FSP1 remains a mystery in mitochondria damage in NP cells in this study. Although we indeed noticed that mitochondrial potential was reversed upon high FSP1 expression, it was difficult to clarify whether the appearance of cristae break and aberrant membrane structure was associated with FSP1 transposition. In general, these dilemmas largely motivate subsequent studies to reveal the manifestations of FSP1 in IDD.

Taken together, this study uncovers the functions of FSP1 during IDD through TNFα-reliant caspase 3-dependent apoptosis. This finding provides a novel target for molecular mechanisms for the treatment of IDD.

### Conflict of interests

The authors have declared that no competing interest exists.

### Funding

This work was supported in part by the National Natural Science Foundation of China (No. 81874022 and 82172483 to Xinyu Liu; No. 82102522 to Lianlei Wang), Key R&D Project of Shandong Province (China) (No. 2022CXGC010503 to Xinyu Liu), Shandong Natural Science Foundation (No. ZR202102210113 to Lianlei Wang), Shandong Province Taishan Scholar Project (No. tsqn202211317 to Lianlei Wang) and National High Level Hospital Clinical Research Funding (No. 2022-PUMCH-D-004).
