# Supplementary material for: TNFα-reliant FSP1 up-regulation promotes intervertebral disc degeneration via caspase 3-dependent apoptosis
Source: Genes Dis. 2024 Feb 28;12(1):101251. doi: 10.1016/j.gendis.2024.101251 (PMC11565395; doi:10.1016/j.gendis.2024.101251)
Supplement: Multimedia component 1 [file mmc1.docx]

**TNFα-reliant FSP1 upregulation promotes intervertebral disc degeneration via Caspase 3 dependent apoptosis**

Cheng Qiu et al.,

**Supplementary figure S1 and Table S1-2.**

**
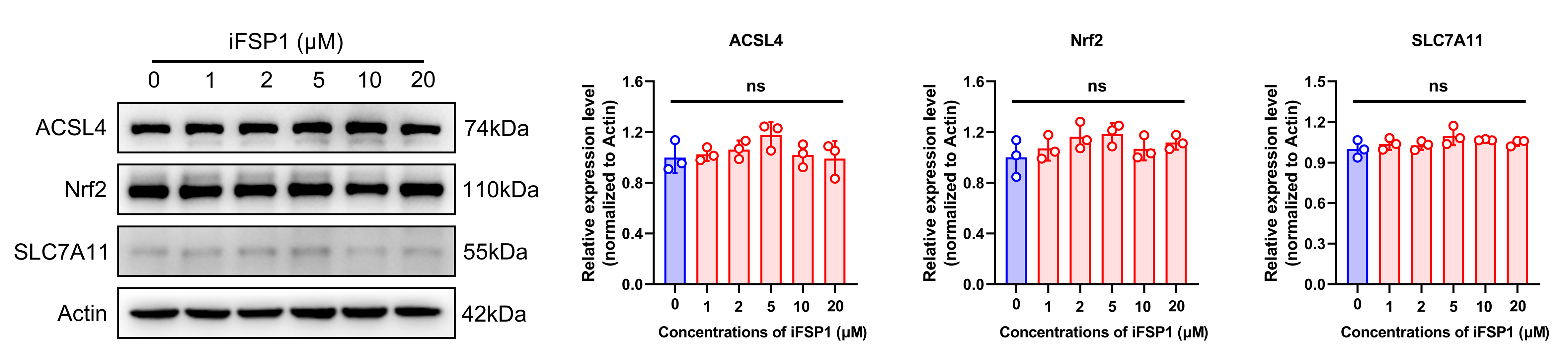
**

**Figure S1.** Western blot of ACSL4, Nrf2, and SLC7A11 expression as well as related quantifications after different iFSP1 (0, 1, 2, 5, 10, 20μM) concentrations treatment.

**Supplementary Table S1. Summary of clinical and demographic features of patients.**

| **Subject Number** | **Gender** | **Age** | **Diagnosis** | **Level** | **Pfirrmann grading** |
| --- | --- | --- | --- | --- | --- |
| **Control group (Grade I-II)** | | | | | |
| C1 | Male | 19 | Spondyloptosis | L4/L5 | I |
| C2 | Male | 52 | Spinal stenosis | L4/L5 | II |
| C3 | Male | 33 | Spinal stenosis | L5/S1 | II |
| C4 | Female | 5 | Hemivertebrae | L1/L2 | I |
| C5 | Female | 15 | Isthmic spondylolisthesis | L5/S1 | II |
| C6 | Male | 3 | Spinal trauma | T1/T2 | I |
| C7 | Male | 21 | Spinal stenosis | L4/L5 | II |
| **Degenerative group (Grade IV-V)** | | | | | |
| D1 | Male | 33 | Disk herniation | L5/S1 | IV |
| D2 | Female | 30 | Disk herniation | L5/S1 | IV |
| D3 | Male | 49 | Disk herniation | L4/L5 | V |
| D4 | Female | 76 | Disk herniation | L4/L5 | V |
| D5 | Male | 47 | Disk herniation | L4/L5 | IV |
| D6 | Female | 16 | Disk herniation | L4/L5 | IV |
| D7 | Female | 63 | Spinal stenosis | L4/L5 | V |
| D8 | Male | 27 | Disk herniation | L3/L4 | IV |
| D9 | Female | 52 | Disk herniation | L5/S1 | IV |
| D10 | Male | 61 | Spondylolisthesis | L4/L5 | V |
| D11 | Male | 30 | Disk herniation | L4/L5 | IV |
| D12 | Female | 31 | Disk herniation | L4/L5 | V |
| D13 | Female | 71 | Spinal stenosis | L5/S1 | V |
| D14 | Male | 66 | Spinal stenosis | L4/L5 | IV |
| D15 | Male | 52 | Disk herniation | L5/S1 | V |
| D16 | Female | 58 | Spinal stenosis | L4/L5 | V |
| D17 | Male | 35 | Disk herniation | L4/L5 | IV |
| D18 | Male | 74 | Spinal stenosis | L3/L4 | V |

**Supplementary Table S2. Antibodies used for Western Blots.**

| **Name** | **Source** | **Catalog number** | **Dilution** |
| --- | --- | --- | --- |
| FSP1 | Proteintech | 20886-1-AP | 1: 1000 |
| GPX4 | Abcam | ab125066 | 1: 2000 |
| ACSL4 | Proteintech | 22401-1-AP | 1: 1000 |
| Nrf2 | Proteintech | 16396-1-AP | 1: 2000 |
| SLC7A11 | Proteintech | 26864-1-AP | 1: 1000 |
| TFRC | Abcam | ab214039 | 1: 1000 |
| FPN | Proteintech | 26601-1-AP | 1: 1000 |
| DMT1 | Abcam | ab55735 | 1: 1000 |
| OPA1 | Proteintech | 66583-1-Ig | 1: 1000 |
| Drp1 | Proteintech | 12957-1-AP | 1: 1000 |
| Mfn1 | Proteintech | 13798-1-AP | 1: 1000 |
| Mfn2 | Proteintech | 12186-1-AP | 1: 1000 |
| Bax | Abmart | PA3143 | 1: 1000 |
| Bcl2 | Abmart | T40056 | 1: 1000 |
| Caspase 3 | Proteintech | 19677-1-AP | 1: 1000 |
| NF-κB p65 | Proteintech | 66535-1-Ig | 1: 1000 |
| IκB-α | Cell Signaling Technology | #4814 | 1: 1000 |
| pIκB-α | Cell Signaling Technology | #2859 | 1: 1000 |
| β-Actin | Servicebio | GB15003 | 1: 2000 |
| α-Tubulin | Proteintech | 11224-1-AP | 1: 3000 |
